# Swept-Source OCT Angiographic Characteristics of Treatment-Naïve Nonexudative Macular Neovascularization in AMD Prior to Exudation

**DOI:** 10.1167/iovs.62.6.14

**Published:** 2021-05-13

**Authors:** Mengxi Shen, Qinqin Zhang, Jin Yang, Hao Zhou, Zhongdi Chu, Xiao Zhou, William Feuer, Xiaoshuang Jiang, Yingying Shi, Luis de Sisternes, Mary K. Durbin, Ruikang K. Wang, Giovanni Gregori, Philip J. Rosenfeld

**Affiliations:** 1 Department of Ophthalmology, Bascom Palmer Eye Institute, University of Miami Miller School of Medicine, Miami, Florida, United States; 2 Department of Bioengineering, University of Washington, Seattle, Washington, United States; 3 Research and Development, Carl Zeiss Meditec, Inc., Dublin, California, United States

**Keywords:** age-related macular degeneration, nonexudative macular neovascularization, swept-source OCT angiography

## Abstract

**Purpose:**

To investigate the characteristics of treatment-naïve nonexudative macular neovascularization (MNV) in age-related macular degeneration before the onset of exudation using swept-source optical coherence tomography angiography.

**Methods:**

MNV area, choriocapillaris (CC) flow deficits (FDs), vessel area density (VAD), vessel skeleton density (VSD), retinal pigment epithelial detachment (PED) volume, mean choroidal thickness (MCT), and choroid vascularity index (CVI) measurements were assessed at two visits prior to exudation. We compared measurements made at the second visit and the rate of change between visits in eyes with and without exudation. The differences in these parameters between eyes with and without subsequent exudation were summarized with 95% confidence intervals (CIs).

**Results:**

Twenty-one eyes with nonexudative MNV were identified and followed. Nine eyes developed exudation, and 12 eyes did not develop exudation. Differences between these groups of eyes for all parameters tended to be small, and the 95% CIs largely ruled out any substantial differences. Overall, eyes with exudation had 24% smaller VAD, 20% smaller VSD, and 33% smaller PED volume measurements. No noteworthy differences were observed for MNV area, CC FDs, MCT, or CVI measurements**.**

**Conclusions:**

The onset of exudation was correlated with lesions having less vascularity and smaller PED volume measurements, but measurements of MNV area, CC FDs, MCT, and CVI were not correlated with near-term exudation. Investigations are ongoing to further explore these and other anatomic changes as harbingers of near-term exudation.

In eyes with dry age-related macular degeneration (AMD), treatment-naïve nonexudative macular neovascularization (MNV) can be detected before the onset of exudation by using indocyanine green angiography and optical coherence tomography angiography (OCTA) imaging.^1^^–^^4^ By definition, these subclinical, nonexudative neovascular lesions are not associated with detectable macular fluid on OCT imaging or with leakage during fluorescein angiography. Most of these lesions are categorized as type 1 MNV, but subclinical type 3 MNV can be detected as well using OCTA.^5^^,^^6^ The type 1 nonexudative MNV can also be detected on structural OCT images by identifying the presence of a double-layer sign or a shallow irregular retinal pigment epithelial detachment (PED).^7^^,^^8^

OCTA is the non-invasive imaging method of choice for the detection of these treatment-naïve nonexudative neovascular lesions.^9^ We have shown that swept-source OCTA (SS-OCTA) appeared to be comparable to indocyanine green angiography for the detection of these lesions.^1^ Using SS-OCTA, we followed the natural history of 227 AMD patients with exudative disease in one eye and nonexudative disease in their fellow eye. We showed that the 2-year risk of exudation in the eye with nonexudative AMD increased nearly 14-fold if treatment-naïve nonexudative MNV was detected compared with eyes in which no MNV was found.^3^ Whether these subclinical lesions should be treated prophylactically with intravitreal injections of vascular endothelial growth factor (VEGF) inhibitors to prevent the onset of exudation has been a topic of debate. We advocate close clinical follow-up without treatment until symptomatic exudation develops. A prospective study in which high-risk eyes with nonexudative AMD were randomized to receive anti-VEGF therapy or sham injections has been conducted. The results published to date have shown no benefit from anti-VEGF therapy every 3 months in preventing exudation or vision loss.^10^

Although some nonexudative neovascular lesions can stay dry for many years, other lesions progress to develop symptomatic exudation.^4^^,^^11^^–^^13^ Because early detection of exudative MNV produces better anti-VEGF treatment outcomes, we recommend close follow-up in clinic and home monitoring for these patients.^14^ In our previous natural history study, we attempted to determine whether anatomic changes could serve as harbingers of near-term exudation, as several groups had reported that morphological changes detected by OCTA imaging of previously treated MNV could reveal patterns of anatomic changes that appeared to predict near-term recurrent exudation.^15^^–^^17^ We investigated the association between MNV size and the rate of change in lesion size measurements just prior to exudation, but no obvious association between lesion size and the onset of exudation was identified. Other ocular parameters that might predict impending exudation include changes in choriocapillaris (CC) perfusion around the MNV, vessel area density (VAD), vessel skeleton density (VSD), volumetric measurement of the associated PED, and surrogate measures of choroidal perfusion such as choroidal thickness (CT) and the density of the choroidal vascular volume, also known as the choroidal vascularity index (CVI).^18^ We were encouraged to investigate these different parameters in eyes with treatment-naïve nonexudative AMD because a number of studies using OCTA imaging of eyes with established exudative MNV have reported that exudation was associated with an increase in MNV size, CC flow deficits (FDs) associated with MNV, higher increase in VAD per year and VSD per year, and changes in the CT and the CVI.^19^^–^^27^

To identify potential biomarkers that might indicate near-term future exudation, we measured the changes in MNV size, CC FDs outside the neighborhood of nonexudative MNV regions, VAD, VSD, PED volume, CT, and CVI in treatment-naïve nonexudative neovascular AMD eyes and investigated whether any of these measurements correlated with the onset of exudation.

## Methods

Patients with AMD were enrolled in a prospective, observational OCT imaging study at the Bascom Palmer Eye Institute. The institutional review board of the University of Miami Miller School of Medicine approved the study, and all patients signed an informed consent for this prospective OCT study. The study was performed in accordance with the tenets of the Declaration of Helsinki and complied with the Health Insurance Portability and Accountability Act of 1996.

Between April 2016 and June 2019, patients with AMD with treatment-naïve, nonexudative MNV were identified and underwent SS-OCTA imaging by the same technician during their follow-up visits. Follow-up visits were determined by routine clinical care and not dictated by a formal protocol. Some patients were followed for bilateral nonexudative AMD, and other patients were seen more frequently due to the need for intravitreal injections in their fellow eyes. The last follow-up visit included in this study was February 10, 2020. Patients were seen usually every 2 to 3 months. Only eyes with subclinical type 1 MNV were included. Eyes with subclinical type 3 MNV and polypoidal choroidal vasculopathy were excluded. Eyes with small neovascular lesions (lesion size at visit 1 < 250 µm in greatest linear dimension or 0.2 mm^2^) were excluded due to the difficulty of measuring these lesions reproducibly. Only lesions fully contained within a 6 × 6-mm scan area were included in this study due to the need for reproducible measurements under and around the lesions. In addition, eyes with geographic atrophy (GA) were excluded due to the difficulty of quantifying the CC within the area of GA. The patients were divided into two groups based on whether the nonexudative MNV developed exudation. If the nonexudative MNV developed exudation, then the last two visits before the visit when exudation was documented were selected for analysis. If the MNV did not develop exudation, then the two consecutive visits at least 6 months before their last follow-up visit were used for analysis to ensure a buffer between the analysis date and possible future exudations. Time of symptomatic exudation in this study was defined as the day the patient received anti-VEGF treatment. The presence of exudation was determined by reviewing individual B-scans to detect intraretinal or subretinal fluid. In this study, the accumulation of fluid under the retinal pigment epithelium (RPE) in the absence of subretinal or intraretinal fluid was not considered evidence of exudation, and anti-VEGF therapy was not offered. The volumes of these PEDs were assessed as described below.

SS-OCTA images were acquired using an instrument (PLEX Elite 9000; Carl Zeiss Meditec, Inc., Dublin, CA) with a swept-source laser source that had a central wavelength of 1050 nm and a scanning speed of 100,000 A-scans per second. The axial resolution of the system is ∼5 µm in tissue, and the lateral resolution is ∼20 µm at the retinal surface. Both 6 × 6-mm and 12 × 12-mm scan patterns were acquired for each patient at all visits. All scans were centered on the fovea and were acquired using FastTrack motion correction software. OCTA images on the PLEX Elite 9000 are generated using the complex optical microangiographic (OMAG^c^) algorithm as previously described.^28^^–^^30^ The en face area measurements of the MNV were obtained by using a previously described validated automated algorithm that detected angiographic flow within the outer retina to CC (ORCC) slab,^31^ which is the slab between the outer retinal boundary and the innermost aspect of the CC. Retinal vessel projection artifacts were removed from the ORCC slab before the analysis.^32^ Images were excluded from this study if the signal strength was less than seven, if there were severe motion artifacts, or if any other pathology or media opacity interfered with imaging of the MNV.

**Figure 1.**
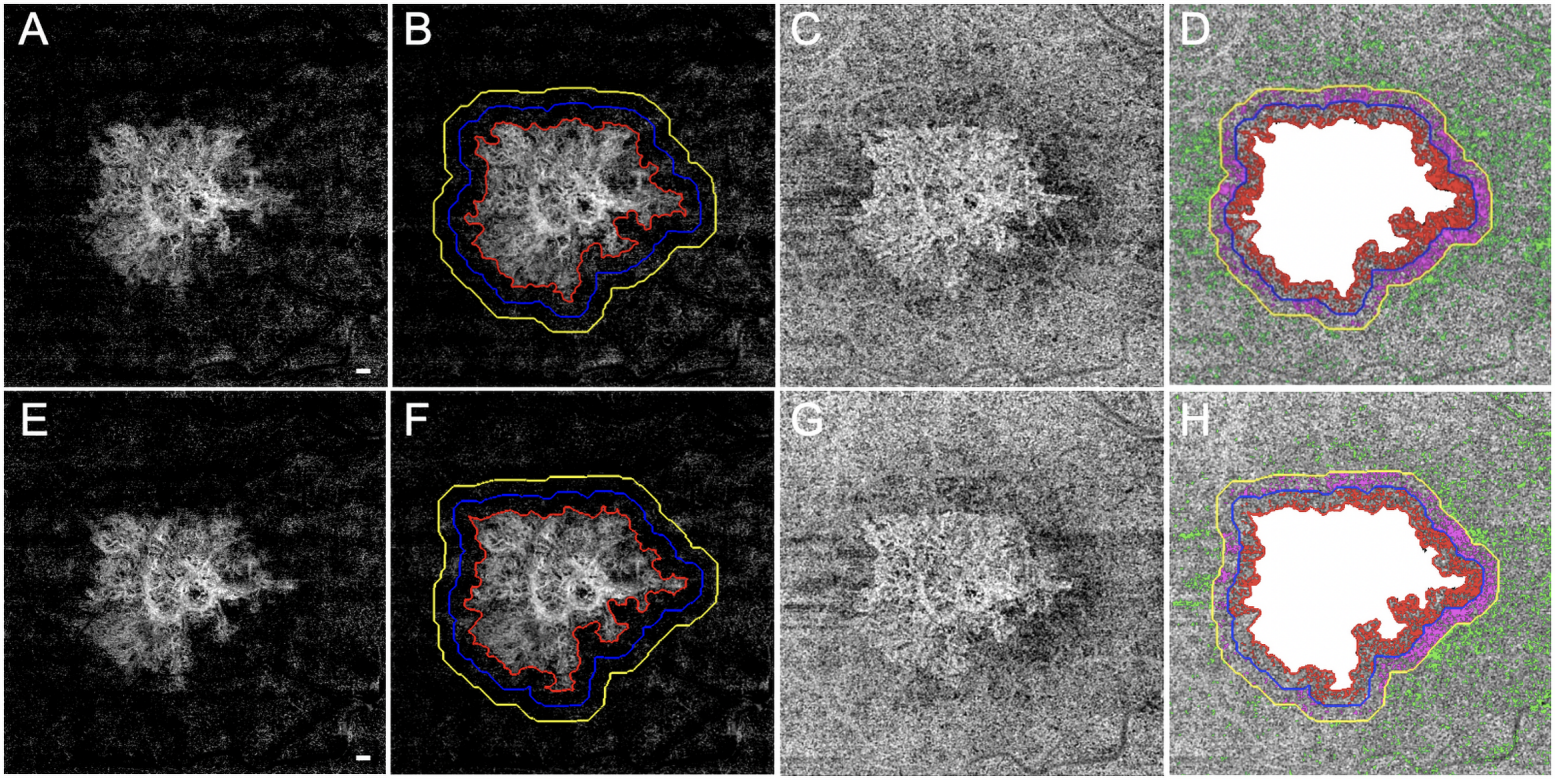
SS-OCTA 6 × 6-mm scans from an eye that did not develop exudation during the follow-up period. The top row images are from the penultimate visit prior to the last visit before the patient was followed for at least 6 consecutive months without exudation. The bottom row images are from the last visit prior to 6 consecutive months without exudation. The interval between these two visits was 84 days. En face flow images of the MNV at visit 1 (**A**) and at visit 2 (**E**). The boundaries of the MNV were outlined in *red* using an automated algorithm with a size of 5.77 mm^2^ at visit 1 (**B**) and 5.74 mm^2^ at visit 2 (**F**). In Region 1 (R1), the 1° rim around MNV (*blue outline*) extends 0 to 300 µm from the margin of the MNV. Region 2 (R2, *yellow outline*) extends 1° farther out, from 300 to 600 µm away from the MNV margin. The third region (R3) represents the scan area excluding the MNV, R1, and R2. En face CC flow images at visit 1 (**C**) and at visit 2 (**G**). (**D**, **H**) Global thresholding measurement of CC FDs minus the area of MNV (*white*). The CC FDs in R1 are color coded in *red*, the CC FDs in R2 are color coded in *purple*, and the CC FDs in R3 are color coded in *green*. At visit 1 (**D**), CC R1 FD% = 31.66%, CC R2 FD% = 23.52%, CC R3 FD% = 7.72%, and CC FD% excluding MNV = 12.17%. At visit 2 (**H**), CC R1 FD% = 27.70%, CC R2 FD% = 23.35%, CC R3 FD% = 7.45%, and CC FD% excluding MNV = 11.58%. *Scale bar*: 300 µm.

The 6 × 6-mm scans were used to compute the CC FD percentage (FD%) and the average CC FD area measurements around the MNV. Visualization and quantification of the CC were obtained using a 16-µm thick slab with its anterior boundary located 4 µm beneath the Bruch's membrane (BM).^33^ The corresponding en face structural slabs were used to compensate for any signal loss due to the overlying anatomy,^34^ and retinal vessel projection artifacts were removed from the CC slab for more accurate measurements (Figs. 1C, G).^32^ CC FD% measurements were obtained from the compensated CC en face flow images and quantified using a global thresholding method.^34^ After thresholding, any FDs with an equivalent diameter smaller than 24 µm were removed, as they were smaller than the average normal intracapillary distance and most likely represented speckle noise.^35^ The CC FD% was defined as the percentage of pixels representing flow deficits relative to all the pixels within a given region. The average CC FD area was defined as the average area of individual FDs within a given region. CC FD% and the average CC FD area were quantified in four different regions around the MNV. Region 1 (R1) represented a 1° field-of-view rim around the MNV; 1° corresponds to a distance of 300 µm (region between the red circle and the blue circle in Figs. 1B, D, F, H). Region 2 (R2) was an additional 1° field-of-view rim extending 300 to 600 µm from the margin of the MNV (region between the blue circle and the yellow circle). Region 3 (R3) included the remaining area when excluding the MNV, R1, and R2 regions from the 6 × 6-mm raster scan (region outside the yellow circle). R1 + R2 + R3 represents the total region excluding the MNV. The CC FDs measurements from R1, R2, and R3 are color coded in red, purple, and green, respectively (Figs. 1D, H). The neovascular lesions were considered to be discrete foci and not lobulations from an existing neovascular lesion if there were no obvious connections between the lesions and the lesions were separated by at least 600 µm, which would correspond to non-overlapping R1 (300 µm) regions around the neovascular lesions.

To quantify the vascularity of the MNV, VAD and VSD were measured from the 6 × 6-mm scans. Because the RPE complex is highly light scattering and reduces the quality of the OCTA MNV images for detailed vascular quantification, we specifically modified our previously validated retinal vascular quantification algorithm^36^ that we developed to accommodate this situation in order to measure the vascular parameters within the MNV. The first step involved removing the noise surrounding the MNV lesion by multiplying the MNV binary mask with the en face MNV flow image generated from the ORCC slab. The resulting MNV image was then subjected to the binary vessel extraction. Briefly, a binary vessel map (Figs. 3B, E) was generated by the combined use of a Hessian filter and adaptive thresholding of the MNV lesion. Then, a skeletonized vessel map (Figs. 3C, F) was created in which every vessel, regardless of its size or diameter, was represented by a single pixel line. The VAD was calculated as a unitless ratio of the MNV image area occupied by the vasculature (summation of the white pixels in Figs. 3B, E) relative to the total MNV area. Similarly, VSD was calculated as the ratio of the length occupied by the vasculature (summation of the white pixels in Figs. 3C, F) relative to the total MNV area.

The volumetric measurements of the PEDs were obtained from 6 × 6-mm scans. The legacy RPE segmentation and the BM segmentation (from the multilayer segmentation as provided by the instrument automated segmentation software) were edited on the instrument as needed to ensure appropriate boundary segmentation. The processed OCT datasets were exported from the instruments and uploaded to the Advanced Retina Imaging Network on the Advanced Retina Imaging Network Hub provided by Carl Zeiss Meditec, Inc. The PED volume was calculated using the Advanced RPE analysis v0.9 algorithm. This algorithm uses the edited RPE and BM segmentations from the instrument as the two segmentation boundaries to measure the PED area and volume, and an en face color-coded PED volume map was created, as well. Because some RPE elevations were associated with drusen while others corresponded to the double-layer sign associated with the MNV, the MNV masks generated by using the ORCC slab as previously described above were superimposed on the PED volume maps in order to isolate the PED volume that was specific for the MNV, which is referred to as MNV–PED volume. Square-root transformation of area measurements and cube-root transformation of volume measurements were performed to eliminate the impact of lesion size on the variability of the standard deviation previously determined from test–retest measurements. The advantages provided by using these transformations were previously described.^37^

Mean choroidal thickness (MCT) and CVI measurements were obtained from the 12 × 12-mm structural SS-OCT scans using an automated strategy as previously reported by Zhou et al.^38^ Attenuation compensation was applied to the OCT scans to eliminate the shadowing effect from retinal layers and to enhance the contrast of the choroidal layer.^38^ BM and the choroid-sclera interface were segmented automatically, and color-coded en face choroidal thickness maps were generated based on these segmentations. On SS-OCT structural scans, choroidal vessels typically appear dark, whereas the choroidal stroma appears as a brighter reflective feature. However, in our en face choroidal vasculature maps the grayscale map is reversed,^18^ so that the choroidal vessels appear bright and the stroma appears dark. MCT was calculated as the mean value of the choroidal thickness within a circular region centered on the fovea with a diameter of 5 mm. Choroidal vessels were segmented from the entire choroidal slab using Otsu's global thresholding method.^39^ CVI en face mapping was generated by calculating the occupation of choroidal vessels at each A-scan position, which is the ratio of the number of pixels that belong to choroidal vessels and the number of pixels that belong to the choroid slab in each A-scan. Then CVI was calculated as the ratio of the choroidal vessel volume to the volume of the choroidal slab region within the same 5-mm circle centered on the fovea. The method of MCT and CVI measurements was applied previously to a normal database in which MCT and CVI were quantified in subregions using both 6 × 6-mm and 12 × 12-mm SS-OCT scans.^18^

Statistical analyses were performed using SPSS Statistics 25 (IBM Corporation, Armonk NY, USA). Data were summarized with mean, standard deviation (SD), median, and interquartile range (IQR). In this descriptive study of many different measurements, we limited statistical inference to the calculation of two-sample *t*-test 95% confidence intervals (CIs) around the differences between eyes that did and did not exhibit exudation after visit 2. These 95% CIs allow us to draw conclusions about the likely size of these differences even with relatively small sample sizes. The normality assumption of the *t*-test was assessed with the Shapiro–Wilk test, and for measurements that violated this assumption, we estimated 95% CIs with bootstrap methods using 2000 resamplings per interval.^40^^,^^41^

## Results

A total of 72 eyes from 70 patients with nonexudative MNV were identified using SS-OCTA imaging between April 2016 and June 2019. Among these 72 eyes, 51 eyes from 50 patients were excluded for further analysis for the following reasons: 14 eyes had inadequate follow-up; 14 eyes had GA at the baseline visit; four eyes developed exudation without having completed two prior visits; five eyes had type 3 MNV; one eye had a polypoidal choroidal vasculopathy lesion; five eyes had small neovascular lesions that were difficult to measure in a reproducible manner (lesion size at visit 1 smaller than 0.2 mm^2^); two eyes had neovascular lesions that extended outside the 6 × 6-mm scan area; and six eyes from five patients had macular pathology other than AMD, including multifocal choroiditis, pathological myopia, and a choroidal nevus.

A total of 21 eyes from 20 patients with nonexudative MNV secondary to AMD were followed in this study. Twelve eyes from 11 patients did not develop exudation, whereas nine eyes from nine patients developed exudation. For eyes that did not develop exudation, the last two visits (defined as visit 1 and visit 2) at least 6 months prior to the last follow-up were selected for assessment. The average time between visit 1 and visit 2 was 3.6 ± 2.1 months. For eyes that developed exudation, the last two visits (visit 1 and visit 2) before exudation were selected for assessment, with the average time between these two visits being 2.7 ± 1.7 months. The difference in the intervals between the last two visits for each group was not statistically significant (*P* = 0.19, Mann–Whitney test). In the eyes that did not develop exudation, the last two visits were at least 6 months before the cut-off for this study, with the average interval from visit 2 to the latest nonexudative status check being 14.8 ± 6.5 months (range, 6 to 29.6). For the group that did develop exudation, the average interval from visit 2 to the day of exudation was 2.1 ± 1.3 months (range, 1 to 4.8). Six out of 18 (33%) monofocal eyes developed exudation compared to three out of three (100%) multifocal eyes, a difference that did not reach statistical significance (*P* = 0.063, Fisher's exact test). Of the three multifocal eyes, one eye had two foci and two eyes had three foci.

**Figure 2.**
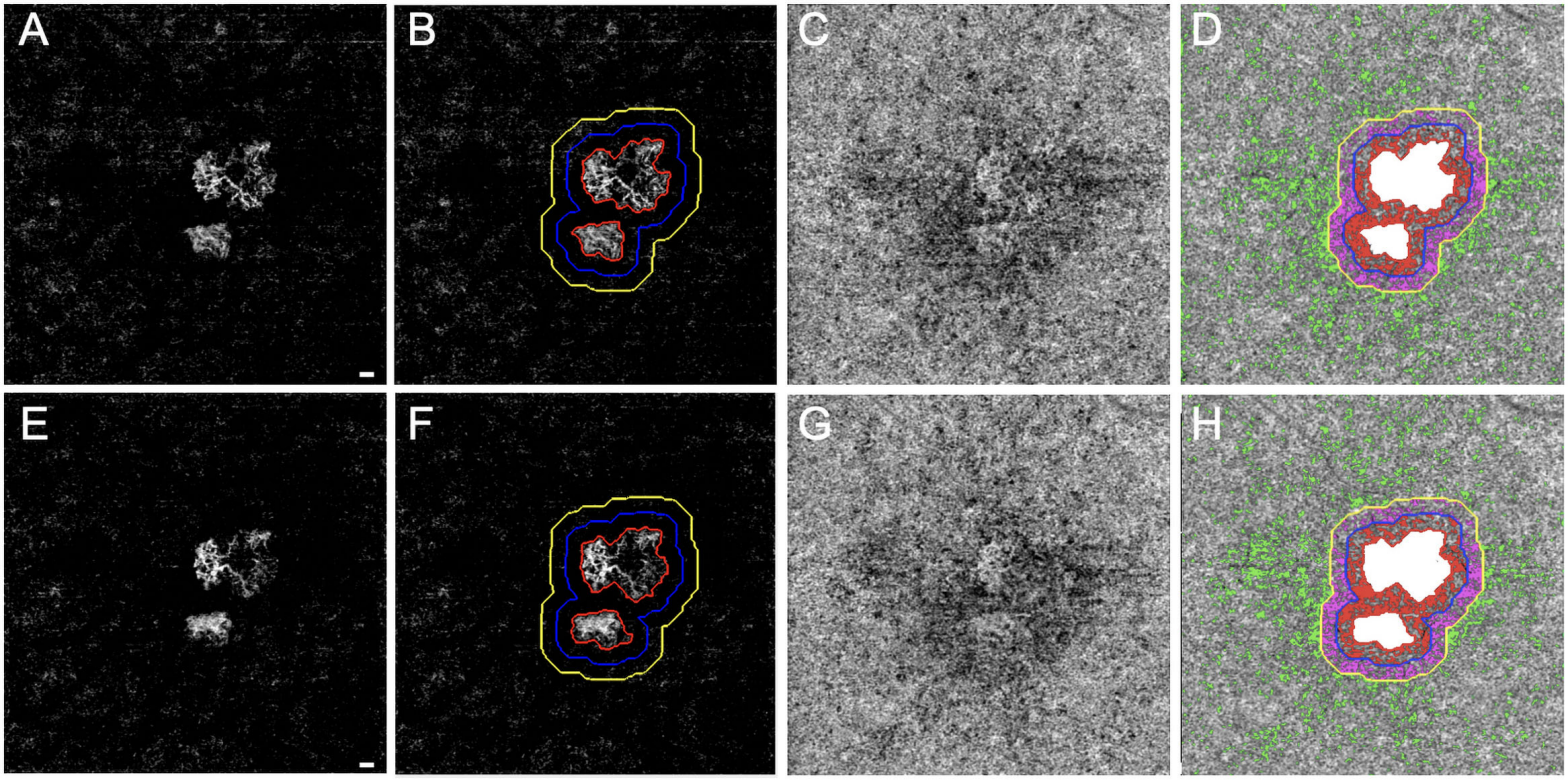
SS-OCTA 6 × 6-mm scans from an eye that developed exudation. The top row images are from the penultimate visit prior to the last visit before exudation, and the bottom row images are from the last visit before exudation. The interval between these two visits was 43 days. En face flow images of the MNV at visit 1 (**A**) and at visit 2 (**E**). The boundaries of the MNV were outlined in *red* using an automated algorithm with a size of 1.45 mm^2^ at visit 1 (**B**) and 1.51 mm^2^ at visit 2 (**F**). The 1° rim around the MNV (R1, *blue outline*) extends from 0 to 300 µm from the margin of the MNV. R2 (*yellow outline*) extends 1° farther out, 300 to 600 µm away from the MNV margin. The third region (R3) represents the scan area excluding the MNV, R1, and R2. En face CC flow images at visit 1 (**C**) and visit 2 (**G**). (**D**, **H**) Global thresholding measurement of CC FDs minus the area of the MNV (*white*). The CC FDs in R1 are color coded in *red*, the CC FDs in R2 are color coded in *purple*, and the CC FDs in R3 are color coded in *green*. At visit 1 (**D**), CC R1 FD% = 41.23%, CC R2 FD% = 35.88%, CC R3 FD% = 9.66%, and CC FD% excluding MNV = 13.17%. At visit 2 (**H**), CC R1 FD% = 43.73%, CC R2 FD% = 36.50%, CC R3 FD% = 9.02%, and CC FD% excluding MNV = 12.80%. *Scale bar*: 300 µm.

Figure 1 shows an eye with nonexudative MNV that did not develop any evidence of exudation during the follow-up period. The top row of images represents the penultimate visit prior to the 6 consecutive months without exudation, and the bottom row of images represents the last visit prior to the 6 consecutive months without exudation. For all eyes without exudation, the average enlargement rate per month of the MNV over these two visits was 0.02 ± 0.1 mm^2^/mo. Figure 2 shows an eye that did develop exudation. The top row of images represents the penultimate last visit before exudation, and the bottom row of images represents the last visit before exudation. In the group that developed exudation, the average enlargement rate of the square-root MNV lesion area over these two visits was 0.01 ± 0.01 mm/mo. Table 1 provides descriptive statistics for eyes with and without exudation. Based on the descriptive statistics shown in Table 1, Table 5 (rows 1–3) presents mean differences in MNV size, square-root MNV size, and CC FD% in the R1 region between eyes that developed exudation and those that did not develop exudation for visit 2 and for the rate of change from visit 1 to visit 2. The 95% CIs around these differences all include zero, which is consistent with the absence of a relationship between these parameters and exudation. However, the marked asymmetry of the CIs around MNV size and square-root MNV size at visit 2, as well as the rate of change in CC R1 FD%, suggests that if a relationship is missed due to our sample size, it is likely to be in the direction of exudation in smaller lesions and those with a faster increase in the CC R1 flow deficit.

**Table 1.** Choriocapillaris Flow Deficit Percentage in Eyes That Did or Did Not Develop Exudation

				Flow Deficit (%) by Region^*^ Mean (SD) Median [IQR]		
Type	Develop Exudation?	MNV Size (mm^2^) Mean (SD) Median [IQR]	SqRt MNV Area (mm) Mean (SD) Median [IQR]	R1	R2	R3
Visit 1	No	3.5 (2.9) 2.3 [5.22]	1.7 (0.8) 1.5 [1.5]	28.6 (13.0) 28.0 [18.1]	25.0 (14.1) 23.1 [11.7]	11.8 (4.5) 12.6 [6.4]
	Yes	2.0 (2.0) 1.4 [2.4]	1.3 (0.7) 1.3 [1.0]	22.3 (9.2) 19.7 [12.2]	24.4 (9.4) 24.8 [16.1]	15.2 (6.0) 13.3 [11.4]
Visit 2	No	3.6 (2.9) 2.6 [5.2]	1.7 (0.8) 1.6 [1.5]	26.4 (14.1) 23.9 [16.9]	24.0 (13.4) 21.3 [8.4]	11.9 (4.0) 12.8 [6.1]
	Yes	2.1 (2.1) 1.5 [2.2]	1.3 (0.7) 1.2 [0.9]	27.2 (13.6) 26.3 [22.7]	25.0 (12.3) 21.5 [17.0]	15.0 (6.1) 14.4 [11.2]
Difference	No	0.0 (0.2) 0.0 [0.3]	0.0 (0.1) 0.0 [0.1]	–2.2 (5.9) –2.9 [7.3]	–1.0 (4.4) 0.2 [6.0]	0.2 (1.3) –0.1 [1.5]
	Yes	0.1 (0.3) 0.0 [0.4]	0.0 (0.2) 0.0 [0.1]	4.9 (9.8) 2.4 [7.2]	0.6 (4.6) 0.6 [4.0]	–0.2 (1.1) –0.3 [1.0]
Rate/month	No	0.0 (0.1) 0.0 [0.1]	0.0 (0.0) 0.0 [0.0]	–0.6 (2.6) –0.5 [2.9]	–0.5 (2.0) 0.0 [1.8]	0.0 (0.5) 0.0 [0.5]
	Yes	0.0 (0.1) 0.0 [0.2]	0.0 (0.0) 0.0 [0.1]	2.0 (3.5) 1.3 [3.4]	0.2 (1.2) 0.4 [2.2]	–0.2 (0.5) –0.2 [0.4]

SqRt, square root.

^*^Region 1, 0 to 300 µm from the margin of the MNV; Region 2, >300 to 600 µm from the margin of the MNV; Region 3, outside Region 1 and Region 2.

**Figure 3.**
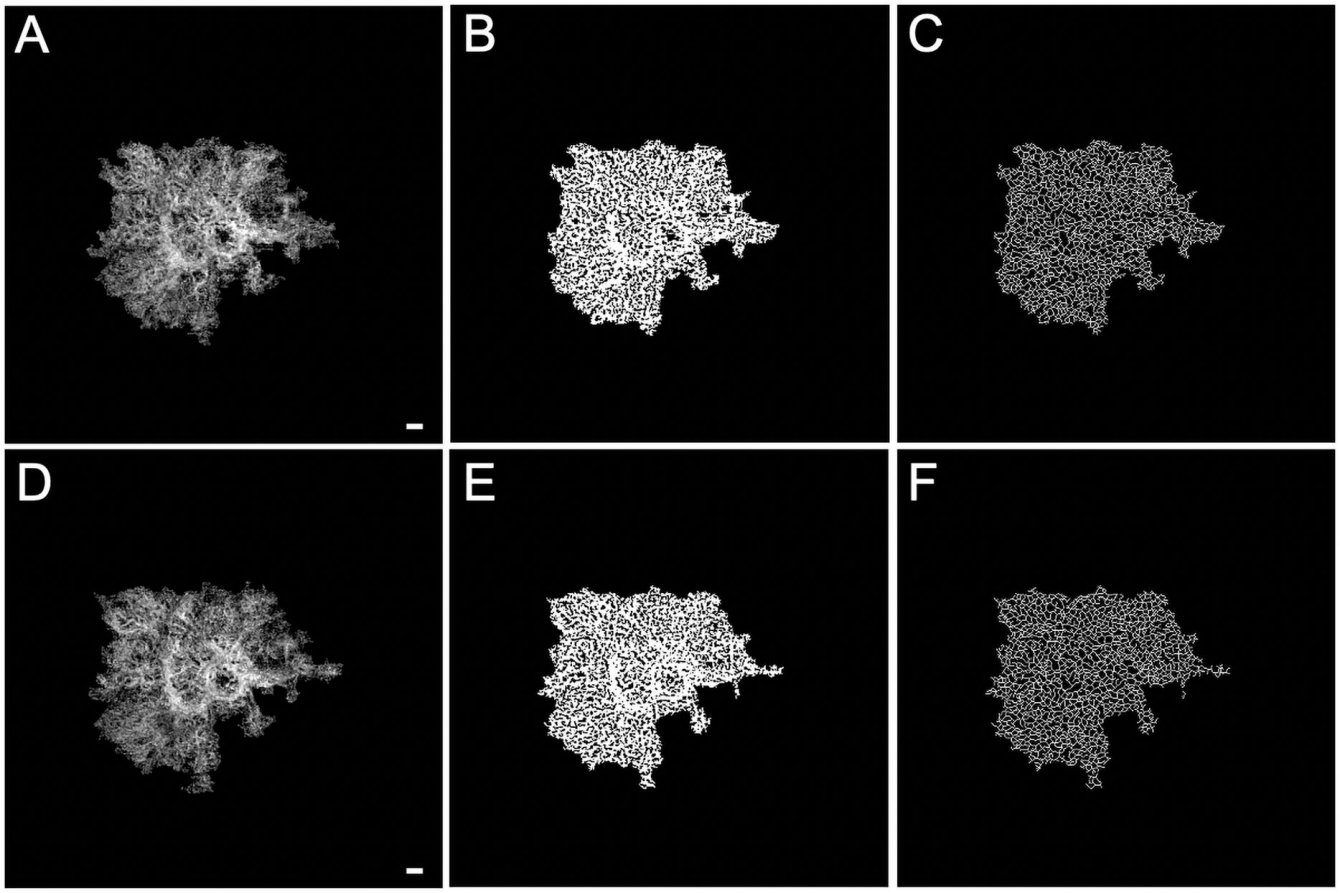
VAD and VSD results from SS-OCTA 6 × 6-mm scans corresponding to the same nonexudative case and visits shown in Figure 1. The top row images are from the penultimate visit prior to the last visit before the patient was followed for at least 6 consecutive months without exudation. The bottom row images are from the last visit prior to 6 consecutive months without exudation. The interval between these two visits was 84 days. (**A**) En face flow images of the MNV at visit 1, and (**D**) images of the MNV at visit 2. (**B**) VAD images at visit 1 = 0.608, and (**E**) VAD images at visit 2 = 0.602. (**C**) VSD images at visit 1 = 0.229, and (**F**) VSD images at visit 2 = 0.229. *Scale bar*: 300 µm.

**Figure 4.**
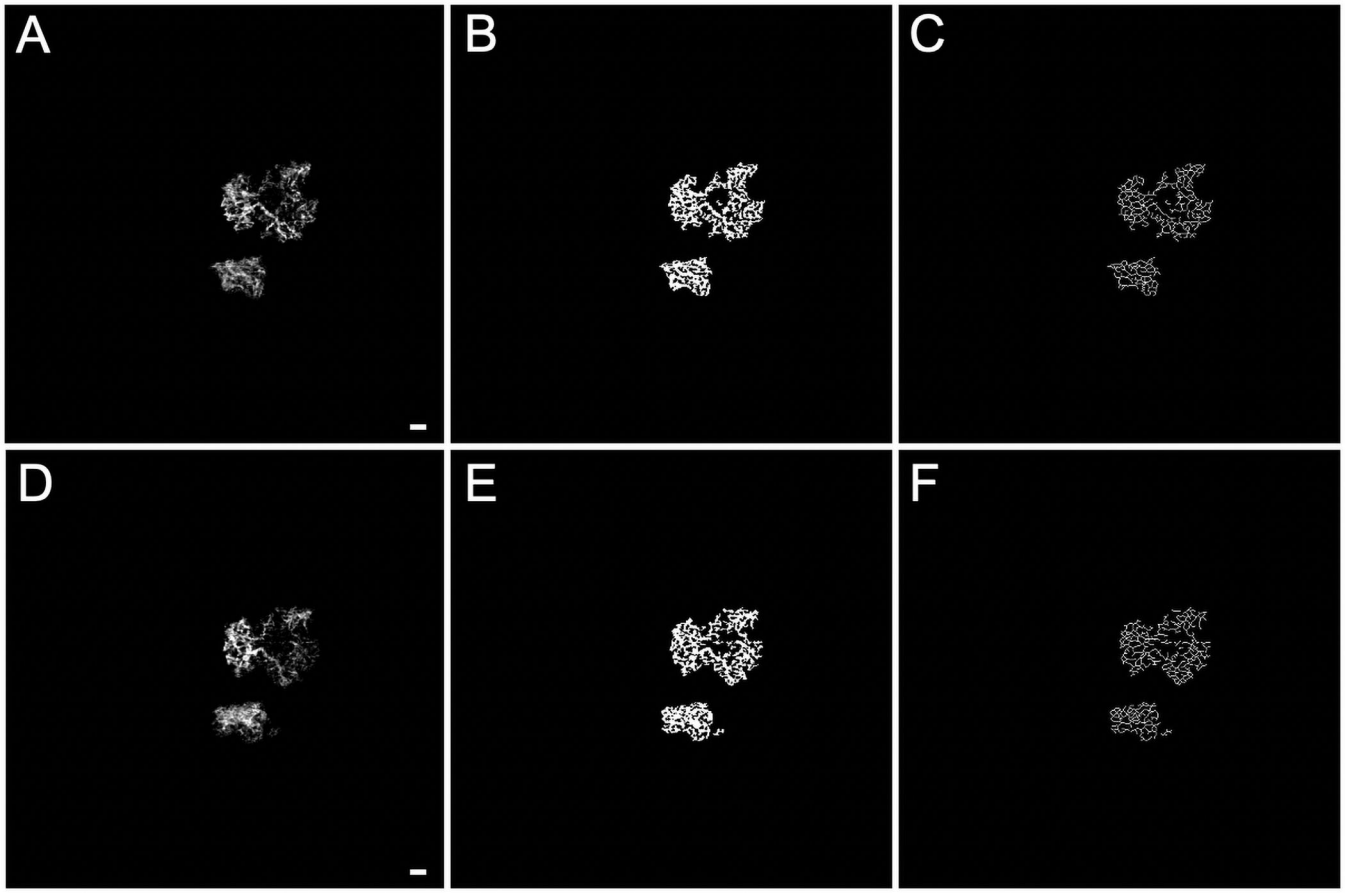
VAD and VSD results from SS-OCTA 6 × 6-mm scans from the same exudative case and the same visits shown in Figure 2. The top row images are from the penultimate visit prior to the last visit before exudation, and the bottom row images are from the last visit before exudation. The interval between these two visits was 43 days. (**A**) En face flow images of the MNV at visit 1, and (**D**) images of the MNV at visit 2. (**B**) VAD images at visit 1 = 0.430, and (**E**) VAD images at visit 2 = 0.417. (**C**) VSD images at visit 1 = 0.159, and (**F**) VSD images at visit 2 = 0.155. *Scale bar*: 300 µm.

VAD and VSD were calculated for the neovascular lesions in all the eyes with nonexudative MNV (Figs. 3, 4). Inspection of Table 2 suggests that both VAD and VSD measurements in the eyes that developed exudation were smaller than those in the eyes that did not develop exudation at both visits. VAD was 18% and 24% smaller in eyes with exudation, and VSD was 11% and 20% smaller in eyes with exudation at visits 1 and 2, respectively. Of note, the 95% CIs on the differences between these groups exclude zero at visit 2 (Table 5, rows 4 and 5), but also suggests that, if differences exist, these differences are small and require further confirmation. There is no indication of a difference in the rate of change between the groups for the VAD, but the VSD appeared to decrease faster in eyes that developed exudation compared with eyes that did not develop exudation. Of note, the 95% CI around the difference in rates excludes zero, but there is the possibility of a very small difference.

**Table 2.** Vessel Area Density and Vessel Skeleton Density in Eyes That Did or Did Not Develop Exudation

Type	Develop Exudation?	VAD Mean (SD) Median [IQR]	VSD Mean (SD) Median [IQR]
Visit 1	No	0.51 (0.07) 0.51 [0.06]	0.18 (0.03) 0.18 [0.04]
	Yes	0.42 (0.09) 0.43 [0.14]	0.16 (0.03) 0.16 [0.04]
Visit 2	No	0.51 (0.05) 0.53 [0.09]	0.19 (0.02) 0.18 [0.03]
	Yes	0.39 (0.10) 0.43 [0.14]	0.15 (0.03) 0.16 [0.04]
Difference	No	0.009 (0.032) 0.001 [0.035]	0.005 (0.012) 0.001 [0.016]
	Yes	–0.031 (0.054) –0.02 [0.06]	–0.008 (0.012) –0.008 [0.016]
Rate/month	No	0.004 (0.014) 0.000 [0.008]	0.002 (0.003) 0.000 [0.005]
	Yes	–0.007 (0.022) –0.010 [0.021]	–0.003 (0.005) –0.003 [0.009]

**Figure 5.**
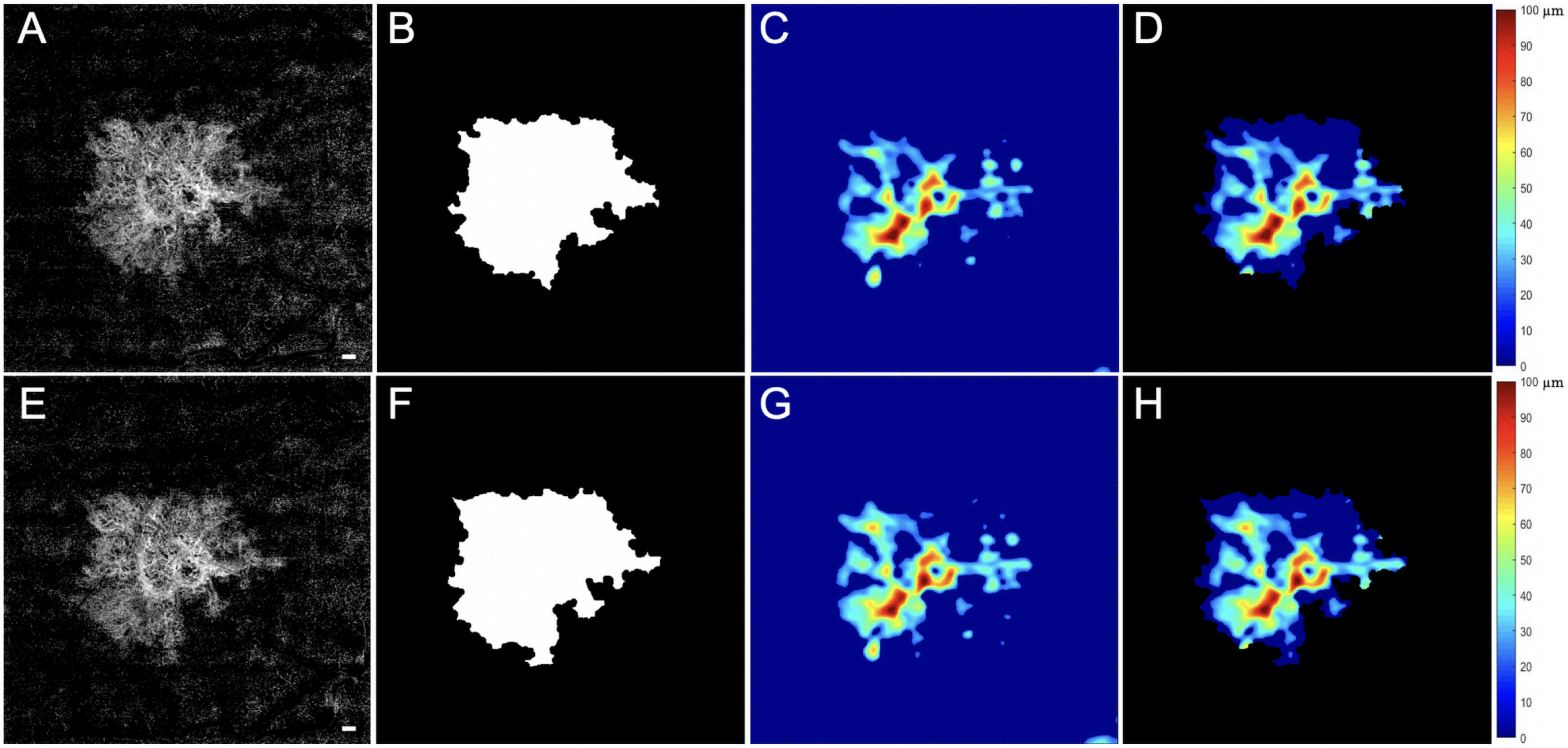
PED volume results from SS-OCTA 6 × 6-mm scans from the same nonexudative case and the same visits shown in Figure 1. The top row images are from the penultimate visit prior to the last visit before the patient was followed for at least 6 consecutive months without exudation. The bottom row images are from the last visit prior to 6 consecutive months without exudation. The interval between these two visits was 84 days. En face flow images of the MNV at visit 1 (**A**) and at visit 2 (**E**). *White* MNV masks at visit 1 (**B**) and at visit 2 (**F**). Total PED volume maps at visit 1 (**C**) = 0.12 mm^3^ and at visit 2 (**G**) = 0.14 mm^3^. MNV–PED volume maps generated by superimposing the MNV mask (**B**, **F**) on the total PED volume maps (**C**, **G**) at visit 1 (**D**) = 0.12 mm^3^ and at visit 2 (**H**) = 0.13 mm^3^. *Scale bar*: 300 µm.

**Figure 6.**
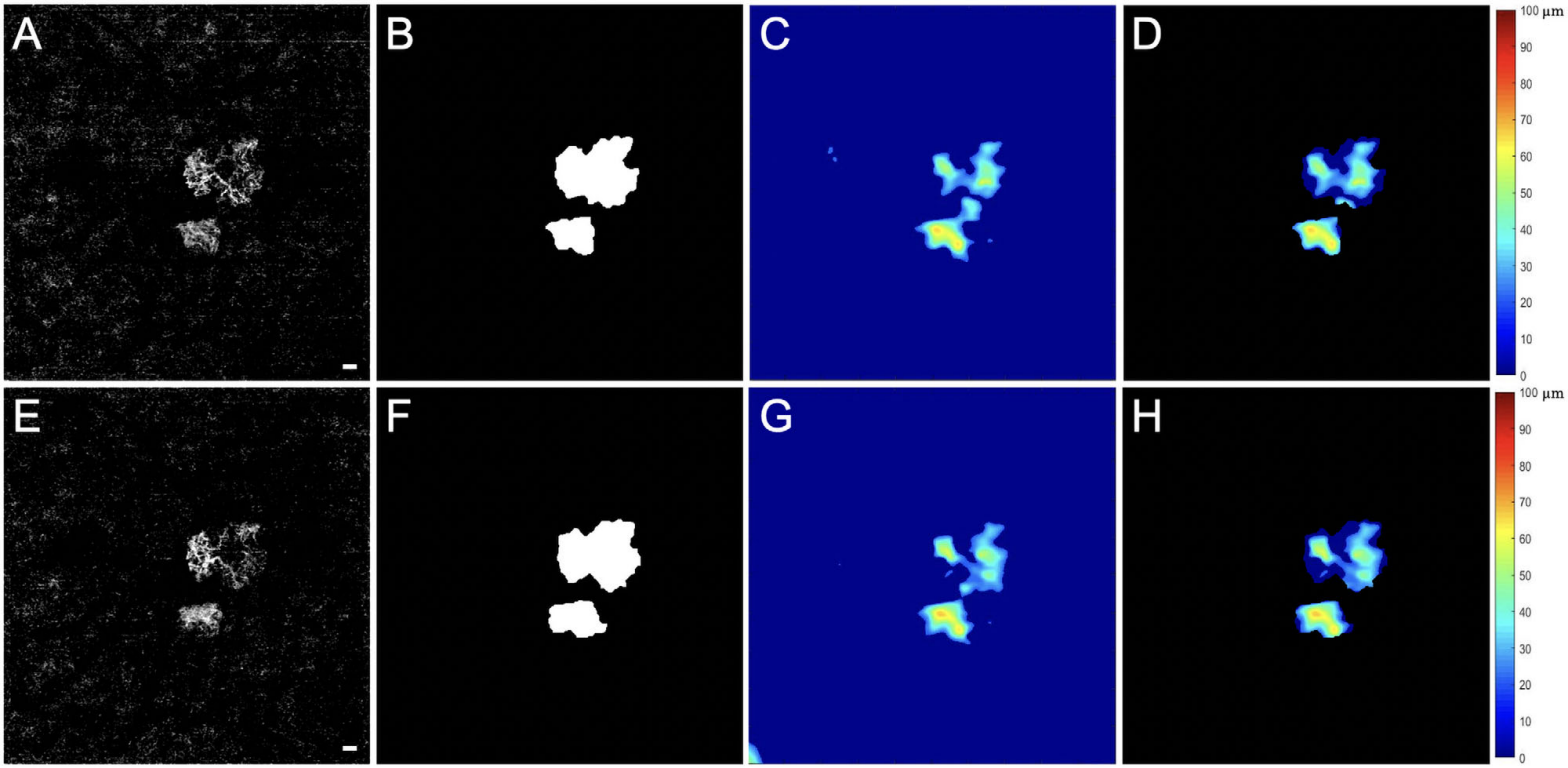
PED volume results from SS-OCTA 6 × 6-mm scans from the same exudative case and the same visits shown in Figure 2. The top row images are from the penultimate visit prior to the last visit before exudation, and the bottom row images are from the last visit before exudation. The interval between these two visits was 43 days. En face flow images of the MNV at visit 1 (**A**) and at visit 2 (**E**). *White* MNV masks at visit 1 (**B**) and at visit 2 (**F**). Total PED volume maps at visit 1 (**C**) = 0.04 mm^3^ and at visit 2 (**G**) = 0.04 mm^3^. MNV–PED volume maps generated by superimposing the MNV mask (**B**, **F**) on the total PED volume maps (**C**, **G**) at visit 1 (**D**) = 0.03 mm^3^ and at visit 2 (**H**) = 0.03 mm^3^. *Scale bar*: 300 µm.

When comparing the area and volume of the MNV–PED measurements between the eyes that did and did not develop exudation (Figs. 5, 6), we found that the eyes developing exudation had smaller MNV–PED volume measurements at both visit 1 and visit 2 (Table 3). Although the 95% CI of the difference at visit 2 excludes zero, it ranges from a very small possible difference to a large one (Table 5, row 6). The 95% CI on rate of change rules out a substantial difference.

**Table 3.** MNV–PED Area and Volume in Eyes That Did or Did Not Develop Exudation

Type	Develop Exudation?	Area (mm^2^) Mean (SD) Median [IQR]	Volume (mm^3^) Mean (SD) Median [IQR]	SqRt Area (mm) Mean (SD) Median [IQR]	CubRt Volume (mm) Mean (SD) Median [IQR]
Visit 1	No	1.57 (1.15) 1.01 [2.27]	0.074 (0.064) 0.050 [0.106]	1.17 (0.48) 1.01 [0.96]	0.381 (0.132) 0.368 [0.242]
	Yes	0.73 (0.84) 0.41 [1.24]	0.028 (0.035) 0.013 [0.047]	0.74 (0.45) 0.64 [0.79]	0.260 (0.114) 0.237 [0.201]
Visit 2	No	1.86 (1.44) 1.16 [2.84	0.088 (0.075) 0.058 [0.144]	1.26 (0.55) 1.08 [1.10]	0.403 (0.143) 0.386 [0.275]
	Yes	0.81 (0.87) 0.65 [1.31]	0.032 (0.041) 0.022 [0.047]	0.78 (0.48) 0.80 [0.83]	0.268 (0.133) 0.282 [0.212]
Difference	No	0.29 (0.38) 0.18 [0.55]	0.014 (0.017) 0.015 [0.022]	0.09 (0.12) 0.06 [0.20]	0.022 (0.024) 0.017 [0.040]
	Yes	0.09 (0.12) 0.07 [0.21]	0.005 (0.009) 0.002 [0.010]	0.04 (0.13) 0.05 [0.23]	0.008 (0.040) 0.016 [0.068]
Rate/month	No	0.12 (0.17) 0.06 [0.24]	0.005 (0.008) 0.004 [0.008]	0.04 (0.05) 0.02 [0.08]	0.008 (0.010) 0.008 [0.010]
	Yes	0.05 (0.06) 0.04 [0.10]	0.003 (0.005) 0.001 [0.005]	0.04 (0.07) 0.02 [0.08]	0.010 (0.019) 0.005 [0.029]

CubRt, cube root.

**Figure 7.**
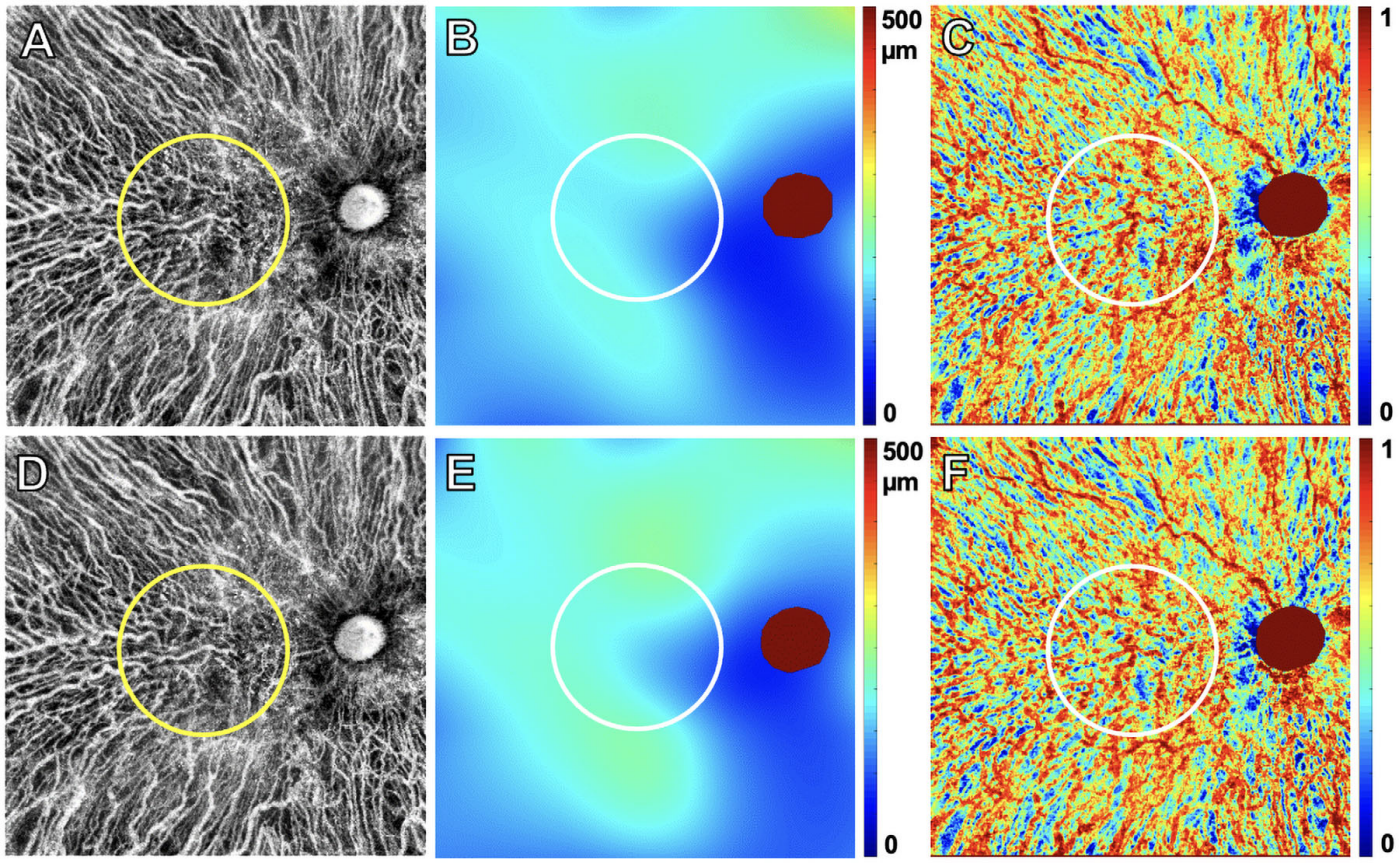
Choroidal vasculature and CT results from SS-OCT 12 × 12-mm scans from the same nonexudative case that did not develop exudation during the follow-up period and the same visits shown in Figure 1. The top row images are from the penultimate visit prior to the last visit before the patient was followed for at least 6 consecutive months without exudation. The bottom row images are from the last visit prior to 6 consecutive months without exudation. The interval between these two visits was 84 days. Choroidal vasculature maps at visit 1 (**A**) and at visit 2 (**D**). The *circle* indicates the 5-mm circle centered on the fovea that was analyzed from these scans. CT maps at visit 1 (**B**) and at visit 2 (**E**); MCT in the 5-mm circle at visit 1 = 169.66 µm and at visit 2 = 182.18 µm. CVI maps at visit 1 (**C**) = 0.62 and at visit 2 (**F**) = 0.62.

**Figure 8.**
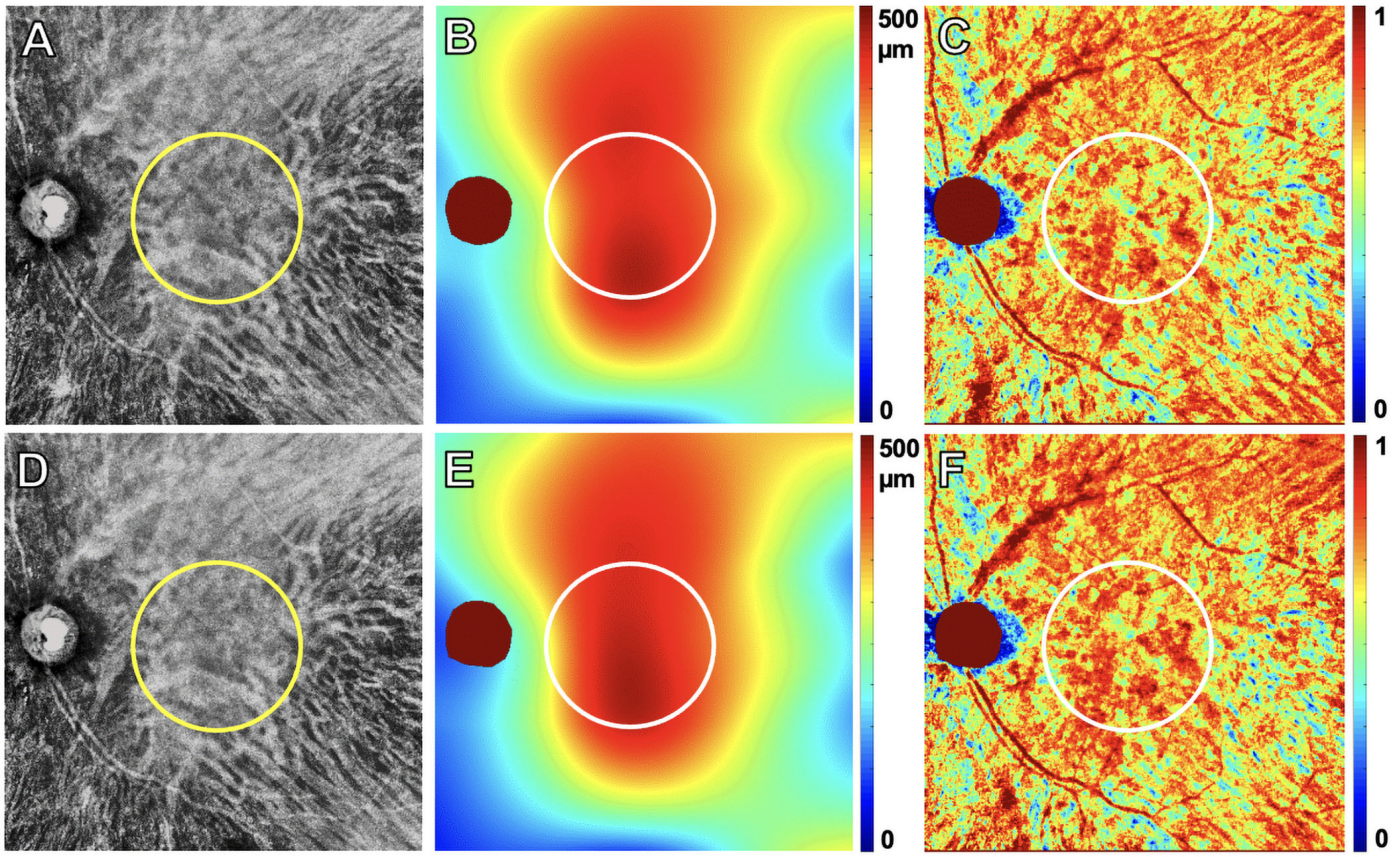
Choroidal vasculature and CT results from SS-OCT 12 × 12-mm scans from the same exudative case that developed exudation and the same visits shown in Figure 2. The top row images are from the penultimate visit prior to the last visit before exudation and the bottom row images are from the last visit before exudation. The interval between these two visits was 43 days. Choroidal vasculature maps at visit 1 (**A**) and at visit 2 (**D**). The *circle* indicates the 5-mm circle centered on the fovea that was analyzed from these scans. CT maps at visit 1 (**B**) and at visit 2 (**E**); MCT in the 5-mm circle at visit 1 = 409.05 µm and at visit 2 = 412.73 µm. CVI maps at visit 1 (**C**) = 0.70 and at visit 2 (**F**) = 0.72.

Figures 7 and 8 show the MCT and CVI measurements within a 5-mm circle centered on the fovea for eyes with and without exudation between visit 1 and visit 2 (see Table 4). Differences between visit 1 and visit 2 are small for these variables (Table 5, rows 7 and 8), and the 95% CI rules out large differences between the visits. Differences between rates of change between eyes with and without exudation are also small, and the 95% CI rules out large differences.

**Table 4.** Mean Choroidal Thickness and Choroid Vascularity Index Between Visit 1 and Visit 2 in Eyes That Did or Did Not Develop Exudation

Type	Develop Exudation?	MCT (µm) Mean (SD) Median [IQR]	CVI Mean (SD) Median [IQR]
Visit 1	No	192.3 (83.9) 173.9 [112.9]	0.619 (0.046) 0.605 [0.081]
	Yes	247.0 (100.4) 279.1 [142.4]	0.633 (0.045) 0.626 [0.052]
Visit 2	No	192.8 (83.9) 183.3 [94.5]	0.614 (0.037) 0.6060 [0.051]
	Yes	244.7 (95.3) 272.9 [136.9]	0.627 (0.042) 0.614 [0.047]
Difference	No	0.5 (10.2) –0.1 [14.6]	–0.005 (0.015) –0.009 [0.023]
	Yes	–2.2 (13.4) 1.2 [19.4]	–0.006 (0.017) –0.005 [0.023]
Rate/month	No	0.7 (3.2) 0.0 [5.9]	–0.002 (0.006) –0.002 [0.008]
	Yes	–1.6 (10.5) 0.4 [6.2]	–0.001 (0.007) –0.003 [0.010]

**Table 5.** A Summary of 95% CIs Around the Mean Difference Between Eyes That Did or Did Not Develop Exudation

	Visit 2			Change Rate Per Month		
	Exudation			Exudation		
Parameter	No Mean (SD)	Yes Mean (SD)	Mean Difference (95% Cl)	No Mean (SD)	Yes Mean (SD)	Mean Difference (95% Cl)
MNV size	3.59 (2.89)	2.09 (2.13)	1.50 (–0.66, 3.51)^*^	0.023 (0.102)	0.038 (0.110)	–0.014 (–0.105, 0.074)^*^
SqRt MNV size	1.74 (0.79)	1.28 (0.71)	0.46 (–0.24, 1.16)	0.01 (0.03)	0.01 (0.04)	0.00 (–0.03, 0.03)
CC R1 FD%	26.4 (14.1)	27.2 (13.6)	–0.8 (–13.6, 12.1)	–0.6 (2.6)	2.0 (3.5)	–2.6 (–5.4, 0.1)
VAD	0.515 (0.054)	0.390 (0.104)	0.125 (0.060, 0.202)^*^	0.005 (0.014)	–0.006 (0.022)	0.011 (–0.006, 0.026)^*^
VSD	0.189 (0.024)	0.149 (0.030)	0.037 (0.012, 0.062)	0.002 (0.003)	–0.003 (0.005)	0.005 (0.001, 0.009)
CubRt MNV–PED volume	0.403 (0.143)	0.268 (0.133)	0.135 (0.007, 0.263)	0.008 (0.010)	0.010 (0.019)	–0.002 (–0.015, 0.012)
MCT	192.8 (83.9)	244.7 (95.3)	–51.9 (−133.9, 30.1)	0.72 (3.19)	–1.64 (10.5)	2.37 (–2.9, 10.2)^*^
CVI	0.614 (0.037)	0.627 (0.042)	–0.013 (–0.050, 0.023)	–0.002 (0.006)	–0.001 (0.007)	–0.001 (–0.007, 0.005)

* 95% CIs on difference were estimated with boot-strapped *t*-test due to failure of normality assumption.

## Discussion

We found that the onset of exudation in eyes with treatment-naïve nonexudative MNV may be associated with neovascular lesions that had lower VAD and VSD measurements, as well as smaller MNV–PED volume measurements, at the visits before exudation. The onset of exudation was not associated with increases in the size of the overall neovascular lesions just prior to exudation or the change in the size of the lesions prior to exudation, so the growth of MNV is not a harbinger of exudation. This is intriguing given the expectation that the onset of exudation would be associated with an increase in the local levels of VEGF, which would be expected to promote lesion vascularity and growth.

It is also noteworthy that the CC FD% measurements and the change in the measurements adjacent to the lesion (R1) were not associated with exudation. However, our attention remains focused on the perfusion of the CC, and this parameter must be studied in a larger sample of patients, as the change in R1 CC FD% was associated with an asymmetric 95% CI. This outcome was of interest because we hypothesized that there would be an increase in CC FD% immediately adjacent to the MNV prior to the onset of exudation or that there would be a difference in CC FD% in the short term when comparing neovascular lesions that remained dry versus lesions that developed exudation. The hypothesis that a difference in CC FD% would exist stems from the current consensus among researchers that MNV arises as a compensatory response to underlying CC impairment, and the ingrowth of MNV is an attempt by the eye to recapitulate the CC in response to growth factors released by distressed RPE and photoreceptors.^42^ Moreover, there have been reports that CC flow impairment has been detected around exudative MNV.^19^^,^^22^ Although we did not show an obvious increase in CC FD% around lesions that were about to develop exudation compared with lesions that remained dry with our current SS-OCTA imaging system, our system was not sensitive enough to detect the CC flow parameters that might predict exudation, such as the relative rates of blood flow within or around the MNV or a change in this blood flow velocity over time. A more advanced research SS-OCTA instrument with a faster scanning rate has been able to detect differences in CC flow around MNV and within MNV^43^^,^^44^ using a 400 kHz SS-OCT instrument and a technique known as variable interval scan time analysis. Rebhun et al.^44^ reported that previously treated neovascular lesions at risk for exudation show an increase in blood flow within the lesion, usually at the margins, and this increased flow precedes recurrent exudation. Another strategy that might prove useful using our current SS-OCTA technology would be to look for surrogate markers that might correlate with this increase in neovascular blood flow. We have explored surrogate markers such as the change in the complexity of the neovascular blood vessels within the lesion or a change in the neovascular volume.^45^ Although the results from these surrogate markers are encouraging, additional studies are needed. Another possibility is that if these parameters are predictive of near-term exudation, we may need to follow these lesions at more closely spaced intervals because the important changes that predict exudation may occur days to weeks before exudation rather than months.

Other surrogates that might reflect changes in the CC, such as changes in the underlying choroidal vasculature, were explored by measuring the MCT and the CVI, but these were not associated with the onset of exudation in our current study. The rationale for a relationship between the risk of exudation and the mean CT and CVI measurements comes from the expected correlations between these two parameters and choroidal blood flow, as both measurements are directly related to the choroidal vascular volume as previously reported by Zhou et al.^18^ If a decrease in choroidal blood flow might cause a decrease in the choroidal vascular volume that could affect both CT and CVI and a decrease in choroidal blood flow might precede a decrease in CC flow (giving rise to an increase in CC FD%), then these choroidal parameters might offer an indirect measure of potential ischemia that might be a trigger for the onset of exudation. Metelitsina et al.^24^ performed a longitudinal study with laser Doppler flowmetry and observed that the development of exudative MNV was associated with lower choroidal circulatory parameters. Therefore, it seemed reasonable that the activity of MNV might be related to the circulation in the choroid and the choroidal parameters we measured. Perhaps a better strategy for addressing this question of choroidal blood flow in nonexudative MNV would involve using laser Doppler flowmetry or a newer technology known as laser speckle flowgraphy that can provide direct measurements of choroidal blood flow.^46^

Our findings were different from the results of Teo et al.,^27^ who found that the eyes with larger baseline MNV size and greater increases in VAD and VSD were more likely to develop exudation. As in our current study, they included 21 eyes with nonexudative MNV, and eight of them progressed to exudation, which was a sample size similar to that of our study. One major difference between our two studies is that they were measuring their changes from the date of exudation to the baseline measurements of the lesions, whereas we employed a more real-world scenario in which we sought to detect near-term changes prior to exudation that would serve as predictors for the near-term onset of exudation. In addition, they measured their baseline MNV lesion size by using a greatest linear diameter parameter rather than the entire lesion area. Based on the quality of the images that they showed, it would appear that they were unable to resolve the entire area of the MNV, which calls into question whether their OCTA instrument was capable of detecting the entire MNV lesion; as a result, the accuracy of their greatest linear diameter measurements is also called into question. Also, based on the poor quality of their OCTA images of the neovascular lesions, we question the reliability of their VAD and VSD measurements, as well.

Contrary to expectations, our data suggest that exudation generally developed in eyes with decreased VAD and VSD measurements and in eyes with smaller MNV–PED volume measurements. Although our findings must be confirmed, it is intriguing to speculate that, if true, then it might reflect a scenario in which the smaller MNV cannot adequately provide the needed nutritional support for the overlying RPE and photoreceptors in the setting of compromised CC perfusion, and the nutritionally deprived RPE and photoreceptors respond by overproducing VEGF. Interestingly, Seddon et al.^47^ observed neovascular buds at the border of the submacular CC atrophy in donor eyes and speculated that these neovascular buds may be a precursor to neovascular disease. If these nonexudative neovascular lesions grow and provide nutritional support for the overlying RPE and photoreceptors, as suggested by a recent histopathological study^26^ and other studies,^48^^,^^49^ then the onset of exudation might be averted, whereas the absence of growth would result in a larger area of poor perfusion that may serve as a stimulus for the onset of exudation. For example, if the nonexudative MNV grows to meet the metabolic needs of the RPE and outer retina, then overexpression of VEGF may not occur, but, if these needs are not met, then the RPE, microglia, and macrophages may be responsible for excessive VEGF production,^50^^,^^51^ resulting in exudation.

Because the sample size of this study was small, we calculated 95% CIs around the differences at visit 2 and the change rates between eyes that did or did not go on to exudation to assess the likely size of differences. Review of these 95% CIs suggests that, despite the small sample size in this study, we can rule out a sizeable difference in the rates of change between eyes with exudation and those without exudation, except that there is a possible faster increase in CC R1 FD% in eyes with exudation. We cannot rule out moderate differences in measurements made at visit 2 between the two groups, although MNV size is unlikely to be larger in the eyes with exudation compared with those without exudation. Although our VAD, VSD, MNV–PED volume, and CC R1 FD% measurements are of interest, we must emphasize that, for all of the parameters studied in this current investigation, our results must be interpreted with caution until they are validated in future studies.

Interestingly, three out of nine eyes that developed exudation had multifocal MNV lesions, whereas none of the eyes that stayed dry had multifocal MNV lesions. That there was no significant difference in the number of lesions between groups suggests that eyes with multifocal MNV may have to be monitored more closely due to the increased risk of exudation. Whether this increased risk of exudation is based on just the increased number of lesions or an increased underlying susceptibility to develop exudation remains to be determined.

The major limitation of our study is its small sample size. Based on this small sample size and the numerous statistical comparisons that we have performed, we present these data as a descriptive study and have refrained from reporting *P* values. We view our results as exploratory and will only be persuaded of their robustness when reproduced with a larger dataset. We remain intrigued with the CC FD% within R1 region. However, even if this region proves to be a useful predictor of exudation in a larger population, it is unlikely that, when measured during the routine follow-up of patients with nonexudative MNV, this CC FD% measurement within R1 would prove to be useful as a definitive near-term predictive metric of exudation at the patient level given the need for a large number of patients to detect this significance, if it exists. Another limitation of our study is the lack of monthly or weekly follow-up visits in the protocol that might have detected changes in the nonexudative MNV that would have occurred closer to the onset of exudation. In our current ongoing prospective natural history study of AMD patients with intermediate AMD or GA, patients are being followed at fixed follow-up intervals of every 3 months; however, this 3-month interval still will not be sufficient to explore whether very near-term changes are correlated with the onset of exudation, but this dataset will allow us to validate the results presented in this report. Although more frequent follow-up visits will be needed to determine if changes might occur within days or weeks prior to exudation, the feasibility of running such a natural history study in an elderly population with AMD is challenging. Our current experience with a schedule of visits every 3 months suggests that most patients would not be willing to return more frequently when no treatment is being offered. With the development of home OCT monitoring, it might be possible to acquire some of the near-term data by remote monitoring. Finally, in order to properly quantify the CC FDs, only 6 × 6-mm scans with a scan density that provided 12-µm spacing between A-scans and B-scans were used for accurate quantitation of FDs. As SS-OCTA scanning speeds increase, we should be able to acquire larger scans with higher scan densities, which will allow us to study CC FDs surrounding larger nonexudative neovascular lesions in the future.

## Conclusions

In summary, we used SS-OCTA imaging to follow eyes with treatment-naïve nonexudative MNV, and we measured various parameters including lesion size, CC FD%, VAD, VSD, MNV–PED volume, MCT, and CVI and compared these measurements between eyes that did or did not develop exudation. Although the eyes that developed exudation seemed to have lower VAD and VSD measurements, smaller MNV–PED volume measurements, and a decrease in VSD over the visits prior to exudation, we interpret these results with caution. The search will continue for characteristic anatomic and blood flow measurements in eyes with nonexudative neovascular lesions that may be useful to predict near-term exudation. Until these markers of near-term exudation are identified and validated, home monitoring and close clinical follow-up are recommended so that anti-VEGF therapy can commence as soon as symptomatic exudation develops.
